# Genetic evidence for predisposition to acute leukemias due to a missense mutation (p.Ser518Arg) in ZAP70 kinase: a case-control study

**DOI:** 10.1186/s12920-024-01961-0

**Published:** 2024-08-07

**Authors:** Khalil Khashei Varnamkhasti, Samire Khashei Varnamkhasti, Atefeh Shahrouzian, Masoomeh Rahimzadeh, Leila Naeimi, Behrouz Naeimi, Sirous Naeimi

**Affiliations:** 1 Department of Medical Laboratory Sciences, Faculty of Medicine, Kazerun branch, Islamic Azad University, Kazerun, Iran; 2 Department of Genetics, College of Science, Kazerun branch, Islamic Azad University, Kazerun, Iran; 3 Department of Medical Laboratory Sciences, Fucalty of Paramedical, Bushehr University of Medical Sciences, Bushehr, Iran; 4 Department of Biology, Zand Institute of Higher Education, Shiraz, Iran

**Keywords:** Mixed phenotype acute leukemia, *ZAP70* gene, rs104893674 (C > A), Missense mutation

## Abstract

**Background:**

The apparent lack of additional missense mutations data on mixed-phenotype leukemia is noteworthy. Single amino acid substitution by these non-synonymous single nucleotide variations can be related to many pathological conditions and may influence susceptibility to disease. This case-control study aimed to unravel whether the *ZAP70* missense variant (rs104893674 (C > A)) underpinning mixed-phenotype leukemia.

**Methods:**

The rs104893674 was genotyped in clients who were mixed-phenotype acute leukemia-, acute lymphoblastic leukemia- and acute myeloid leukemia-positive and matched healthy controls, which have been referred to all major urban hospitals from multiple provinces of country- wide, IRAN, from February 11’ 2019 to June 10’ 2023, by amplification refractory mutation system-polymerase chain reaction method. Direct sequencing for rs104893674 of the *ZAP70* gene was performed in a 3130 Genetic Analyzer.

**Results:**

We found that the AC genotype of individuals with A allele at this polymorphic site (heterozygous variant-type) contribute to the genetic susceptibility to acute leukemia of both forms, acute myeloid leukemia and acute lymphoblastic leukemia as well as with a mixed phenotype. In other words, the *ZAP70* missense variant (rs104893674 (C > A)) increases susceptibility of distinct cell populations of different (myeloid and lymphoid) lineages to exhibiting cancer phenotype. The results were all consistent with genotype data obtained using a direct DNA sequencing technique.

**Conclusion:**

Of special interest are pathogenic missense mutations, since they generate variants that cause specific molecular phenotypes through protein destabilization. Overall, we discovered that the rs104893674 (C > A) variant chance in causing mixed-phenotype leukemia is relatively high.

**Supplementary Information:**

The online version contains supplementary material available at 10.1186/s12920-024-01961-0.

## Background

Acute leukemia is one of the most common types of hematological malignancies which affects all blood cell lineages and is associated with increase in the growth rate and disorganized differentiation of hematopoietic cells [1, 2]. Although acute leukemia usually presents in the myeloid or lymphoid lineages, in rare cases some with no clear evidence of differentiation along a single lineage exhibit two distinct affected cell populations of different lineages [2, 3]. In cases of bi-lineal acute leukemia classified as mixed-phenotype acute leukemia (MPAL), the outcome is worse than both acute lymphoblastic leukemia (ALL) and acute myeloid leukemia (AML) [4] and patients with MPAL have poorer survival as compared with age-matched patients with a single phenotype of acute leukemia thereby MPAL prognosis is largely dependent on age and is likely can give rise by aberrant mechanisms at a genomic scale [5]. Protein tyrosine phosphorylation is a substantial signaling mechanism that controls important cellular processes including cell growth, differentiation, metabolism, and motility by activation of downstream pathways. Aberrant routine tyrosine kinases signaling promotes carcinogenesis, an aberrant which has been observed in association with mutations that permit inhibition of the normal function of protein kinases [6, 7]. Most often, missense mutation is the predominant one which results in amino acid changes in the polypeptide chain [8]. Missense mutations or nonsynonymous substitutions which make single nucleotide variants (SNVs) that manifest as protein variants with a single amino acid variation (SAV), are of particular interest in view of the fact that notwithstanding a single amino acid substitution may cause dramatic structural changes by which protein stability is compromised, or a perturbation that destabilizes binding interfaces in protein to the point of impairing its function [9]. The ζ-associated protein of 70 kDa (ZAP-70) is a cytoplasmic tyrosine kinase that has a role in differentiation by regulating the JAK–STAT3–MYC signaling axis. Additionally, ZAP70 was shown to promote migration and invasion of prostate cancer cells as well as identified as a prognostic marker in colorectal cancer [10–12]. Currently, 19 nonsynonymous single-nucleotide polymorphisms (nsSNPs) in the Single Nucleotide Polymorphism (dbSNP) database have been reported to cause nonsense or missense changes in ZAP70. It is believed that various causes underlie the different types of leukemia, while a genetic factor might reveal common molecular traits that contribute to the development of AML and ALL simultaneously [13, 14]. In this study, from these nsSNPs, we selected rs104893674 with DNA substitution of 1554 C > A and Ser518Arg amino-acid substitution to identify likely common genetic traits between AML and ALL which would help to uncover the points of susceptibility for MPAL.

## Methods

Using a case-control study design, male and female subjects who were diagnosed (depended on complete blood count, peripheral blood film, and flow cytometric immune-phenotyping) with acute myeloid leukemia (n = 94) and acute lymphoblastic leukemia (n = 101), were studied from July 20’ 2021 to June 10’ 2023. The controls for both AML (*n* = 99) and ALL (*n* = 101) groups were selected randomly among the age- and sex-matched general population without neoplastic diseases or patient’s companions at Fars and Isfahan (Iran) urban hospitals. Cases from each subtypes of AML or ALL with secondary leukemia, other hematological disease, accompanied with other systemic malignancies, pregnant and lactating women, subjects with uncontrollable systemic infection were excluded.

Forty-seven blood samples were obtained from cases of nonobvious bi-lineage MPAL presentation that microscopic evaluation of their specimens had revealed the coexistence of two different lineages making up the blast cell population, referred to hospitals across the country (Fars, Isfahan, Tehran, Qom, Khuzestan, Bushehr and Yazd), over a 4-years period (2019–2023). Fifteen samples from patients with MPAL were included (cases expressed mixed phenotypic markers) and thirty-two samples (which unequivocally assigned to a myeloid, B- or T-lymphoid lineage) were excluded from this study. Afterwards, enrollment was stopped before reaching the larger sample size because of poor recruitment (as MPAL is rare and giving an incidence of 0.35 cases/1,000,000 person-years) [15]. Therefore, a minimum ratio of 30% (15 individuals) for this population under 50 ensured representativeness of the sample for quantitative analysis [16]. Also, a total of 23 blood samples were obtained from matched healthy subjects, referring to the same hospitals.

A volume of 10 mL of whole blood of each study participant was collected in blood collection tubes containing EDTA for hematologic analysis. Complete blood count was scored in the Dirui Hematology Analyzer (China). Cytomorphologic abnormalities were recorded using Romanowsky-stained blood film.

Immuno-phenotyping assay was performed through flow cytometry analysis (FACSCalibur flow cytometer, BD Biosciences), following a previously described protocol [17], for identification of leukemia cell subsets by their marker profile. The major markers (either cell surface or intracellular) including B-cell lineage markers (CD19, cCD22 and cCD79a) and T-cell-related markers (CD5, sCD3 and CD4 and CD8) for ALL and myeloid markers (CD13 and MPO) for AML were utilized for the distinction of leukemia cell subsets. Markers of multiple lineages including CD19 and CD79a (B -lymphoid lineage), CD7 (T -lymphoid lineage) and CD13, CD33, and MPO (myeloid lineage) were used to make an accurate diagnosis in MPAL ambiguous cases. FlowJo software (FlowJo LLC) was used to analyze flow cytometry data.

We evaluated the Hardy–Weinberg equilibrium (HWE) by computing the observed genotype frequencies versus expected genotype values for polymorphic locus to check whether the population was in Hardy–Weinberg equilibrium.

We used the windows-based software program QUANTO (Version 1.2.4; University of Southern California, Los Angeles, CA, USA, http://biostats.usc.edu/Quanto.html) to estimate the statistical power of our study for detecting an association of rs104893674 with acute leukemia.

*ZAP70* genetic polymorphism (rs104893674 (C > A)) was assessed by amplification refractory mutation system–polymerase chain reaction (ARMS-PCR), aka Allele-specific polymerase chain reaction (AS-PCR), after peripheral blood samples were collected in EDTA vacutainer tubes and extraction genomic DNA following a salting out technique standard protocol. We used a pair of internal control primers (Beta-actin was used as an internal control) specific for the normal DNA sequence (A pair of control primers which could not amplify mutant DNA at a given locus was used to confirm that the genomic DNA is, in principle, amplifiable) and allele-specific primers designed using Oligo7 software (version 7.54, Molecular Biology Insights Inc., Cascade, CO, USA). (The designed primer sequences reported in Table 1).

**Table 1:** Designed primers for ARMS-PCR reaction

Description	General Characteristics
ZAP70	rs104893674 (Missense)
Type of polymorphism	Single-base C > A
Site of polymorphism	p.Ser518Arg
**PCR primers**	
Forward	5- CGATGAAGGCCATGACCTC -3
Reverse A allele	5- CTTCCGCAAGTTCTCCAGA -3
Reverse C allele	5- CTTCCGCAAGTTCTCCAGC -3
Forward-PCR-Control	5- CCTCTGCACAGTTTGGAC -3
Reverse- PCR-Control	5- TCTGTCCAGCAATCCAGG -3
**PCR conditions**	
Denaturation	94 °C, 5 min
Annealing	58 °C, 40 s
Extension	72 °C, 40 s
No. of cycles	32
**Product Size (bp)**	237 bp
***Beta-actin***	**Internal control**
**PCR primers**	
Forward primer	5- TATCCAGGCTGTGCTATCCCTGTAC -3
Reverse primer	5- CTTGATGAGGTAGTCAGTCAGGTCC -3
**PCR conditions**	
Denaturation	94 °C, 5 min
Annealing	56 °C, 40 s
Extension	72 °C, 40 s
No. of cycles	32
**Product size (bp)**	169 bp

Polymerase chain reaction (PCR) for target DNA amplification was achieved using a final reaction volume of 22 µL composed of, 1 µL template DNA, 11µL of 2× Master Mix Red (Ampliqon), 1 µL of each primer (10 µM), and 5 µL DNase-free water. The PCR cycling conditions were 5 min at 94^◦^C, followed by 32 cycles of 40 s at 94^◦^C, 40 s at 58^◦^C, and 40 s at 72^◦^C, with a final step at 72◦C for 5 min. PCR products were verified on a 2% agarose gel for 10 min and visualized on UV transilluminator. Direct sequencing of PCR amplification products recovered by the GEL/PCR Purification Kit (Favorgen Biotech Corp., Taiwan, China) was analyzed on a 3130 Genetic Analyzer sequencing machine (Applied Biosystems) according to protocol previously described by Ameri et al. [18] and Parhoudeh et al. [16]. Sanger Sequencing PCR reaction components and thermal cycling steps outlined in Tables 2 and 3, respectively. Sequences were analyzed with the CodonCode Aligner V.5.1.5 software (CodonCode Corporation, Centerville, MA, USA).

**Table 2:** Sanger sequencing PCR reaction setup components

Component	Volume per reaction (µl)
Buffer big dye	3
Big-dye enzyme (BDT)	3
Primer	0.33
DW	7.67
DNA	6

**Table 3:** PCR thermal-cycling parameters for sanger sequencing

Cycles	Step	Temperature	Time
1 cycle	Initial Denaturation	94 °C	5 min
	Denaturation	95 °C	15 S
35 cycle	Annealing:		
	rs104893674	60 °C	30 S
	Extension	72 °C	45 S
	Final Extension	72 °C	10 min
	Hold	4 °C	

To analyze the difference between genotype and allele frequencies in two groups, Chi-square testing and logistic regression analyses were used by SPSS software (Version 22.0, SPSS, Inc, Chicago, IL, USA). P-values less than 0.05 were considered statistically significant. Bonferroni corrections were applied to correct for multiple comparisons, and the threshold for statistical significance was set at ≤ 0.05.

## Results

**Fig. 1:**
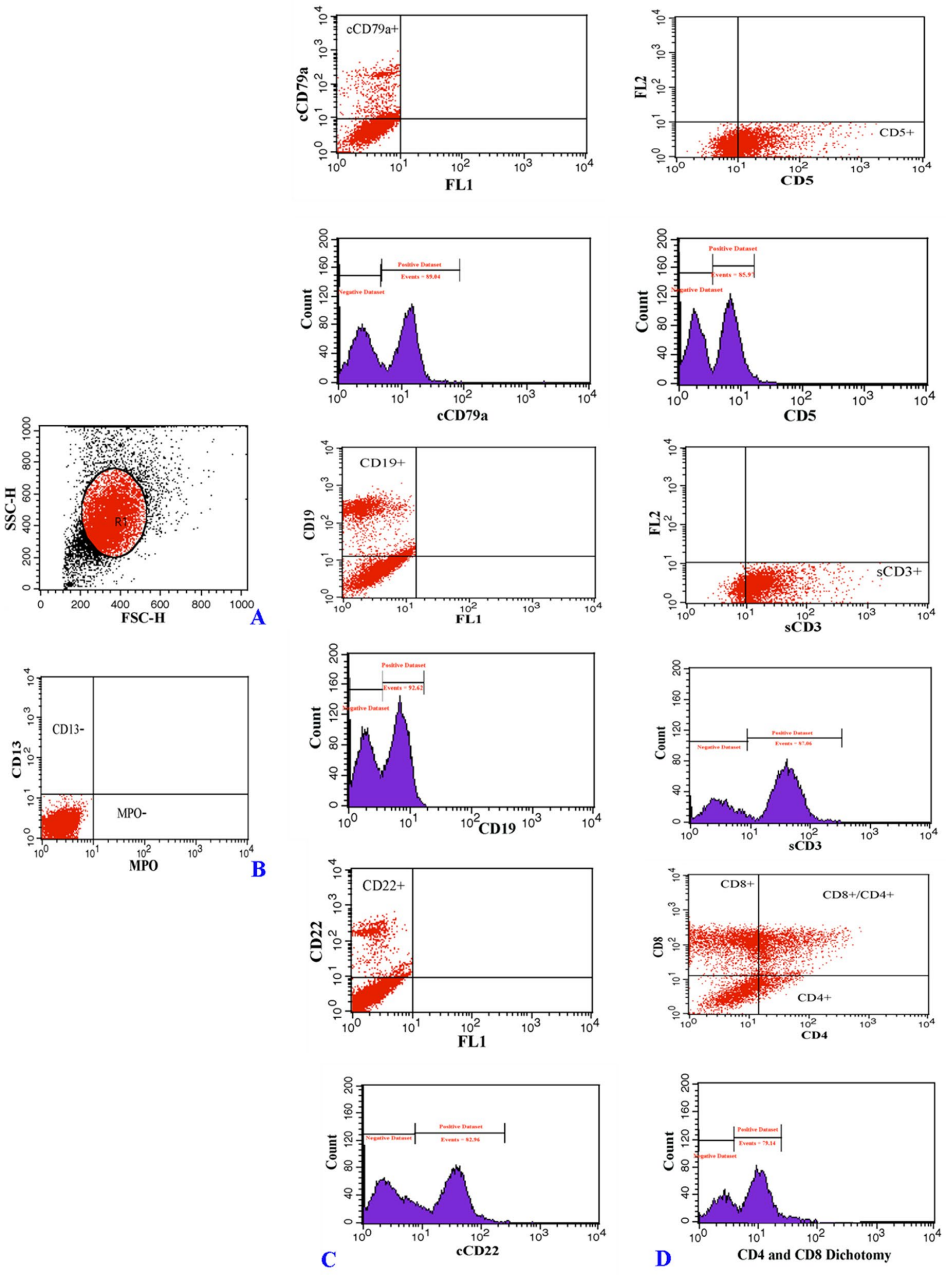
Immuno-phenotyping profile of ALL cases; (**A**) FSC and SSC, morphologic characteristic of blood cells; (**B**) negative expression of acute myeloid leukemia specific markers; (**C**) B-cell lineage acute lymphoblastic leukemia positive for the following markers, cCD79a, CD19, and cCD22 (histograms display a single measurement parameter (Positive dataset)); (**D**) T-cell lineage acute lymphoblastic leukemia positive for the following markers, CD5, sCD3 and co-express of CD4 and CD8 simultaneously (cells double positive or CD4^+^\CD8^+^)

**Fig. 2:**
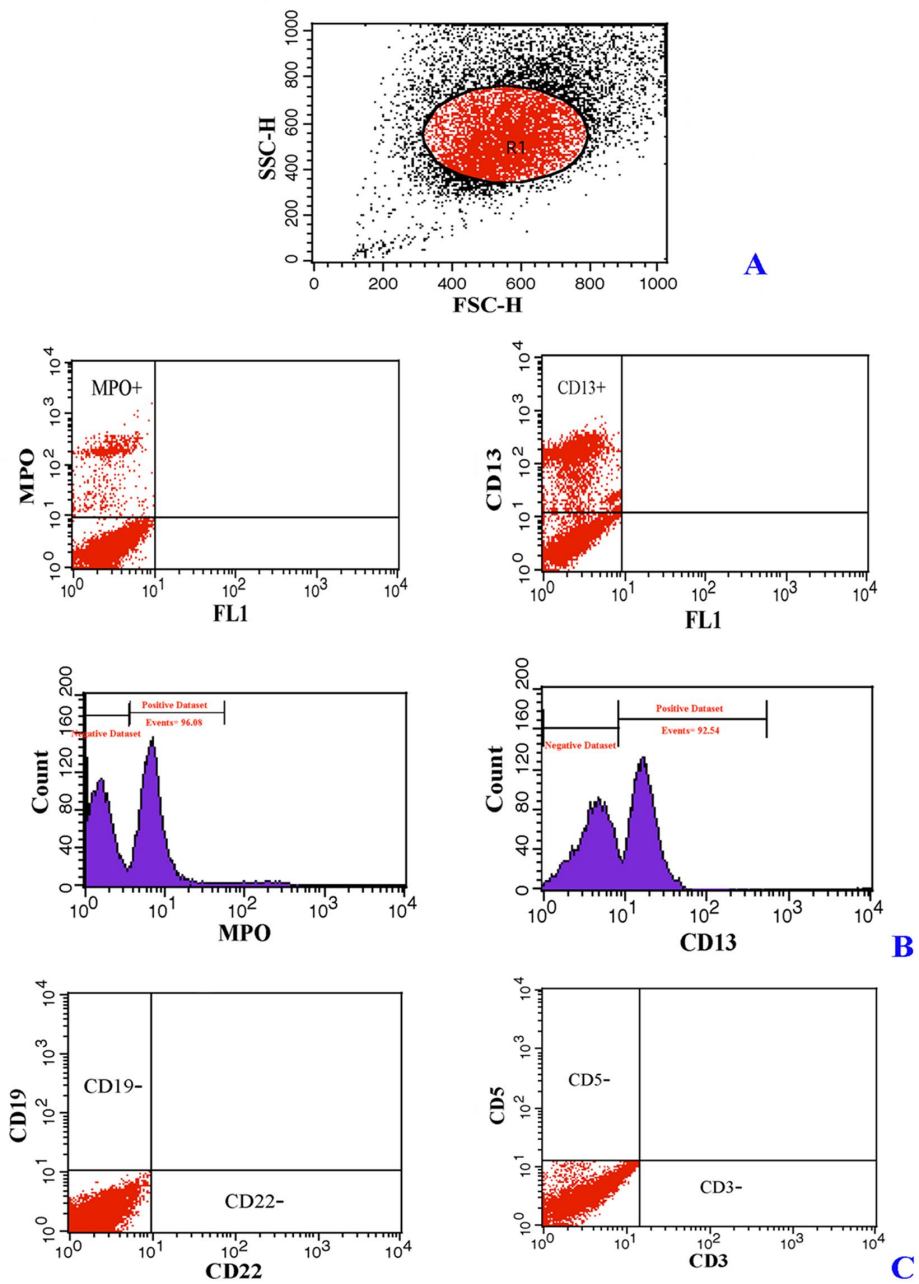
Immuno-phenotyping profile of AML cases; (**A**) FSC and SSC, morphologic characteristic of blood cells; (**B**) myeloid leukemia positive for the MPO and CD13 markers (histograms display a single measurement parameter (Positive dataset)); (**C**) negative expression of acute lymphoblastic leukemia specific markers

**Fig. 3:**
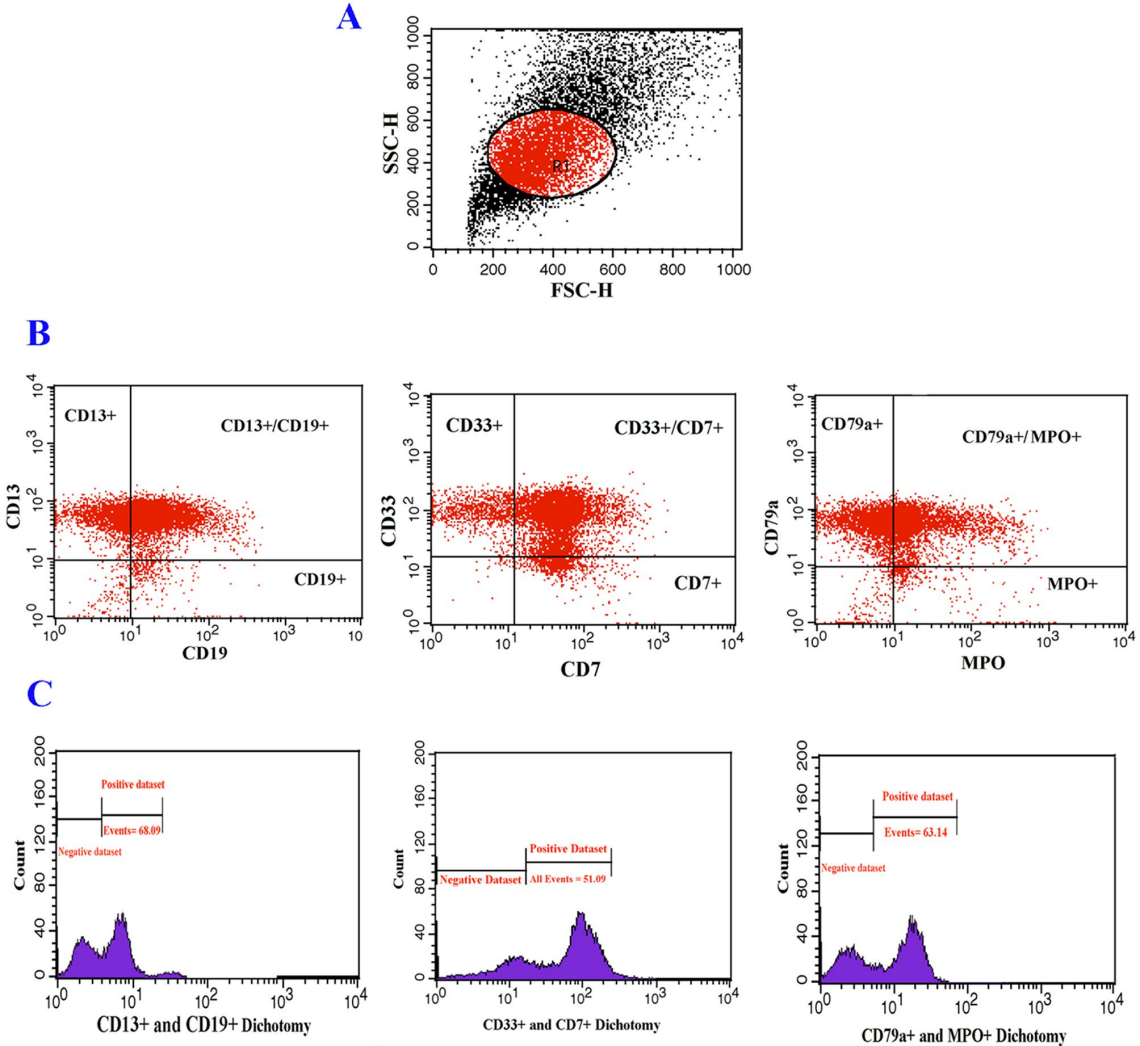
Immuno-phenotyping profile of MPAL cases; (**A**) FSC and SSC, morphologic characteristic of blood cells; (**B**) Positive markers for multiple lineages including, CD19 and CD79a (B -lymphoid lineage), CD7 (T -lymphoid lineage) and CD13, CD33, and MPO (myeloid lineage); (**C**) Histograms display positive datasets

According to immuno-phenotyping findings, common Pre-B ALL was diagnosed in 75% of study’s cases from ALL group who expressed CD19, cytoplasmic- CD22 (cCD22) and CD79a (cCD79a) markers. A total of 25% of cases of this group were represented as T-cell ALL which characteristically expressed CD5, surface CD3 (sCD3) and CD4/CD8 dichotomy markers (Fig. 1). The leukemic cells from all cases in the AML group expressed myeloid markers including CD13 and MoAb anti myeloperoxidase (MPO) (Fig. 2). Immuno-phenotyping results showed that bi-phenotypic cases co-expressed myeloid, B- and T-lymphoid antigens (Fig. 3).

We calculated the genotype frequencies expected versus observed genotype values under traditional HWE test for polymorphic locus to determine whether the population was in Hardy–Weinberg equilibrium. As the deviation from HWE in the polymorphic locus was not significant, the results indicated the presence of HWE in in question population at polymorphic rs104893674 site (Table 4).

**Table 4:** Hardy Weinberg equilibrium

Phenotype	Genotype	Obs. (exp.)	HWE *p*-value
ALL	CC AC AA	37(49) 64(42) 0 (9)	
			0.747
AML	CC AC AA	14 (10) 79 (86) 6 (4)	
			0.608
MPAL	CC AC AA	14(60.8) 6 (26.1) 3 (13.1)	
			0.763

**Fig. 4:**
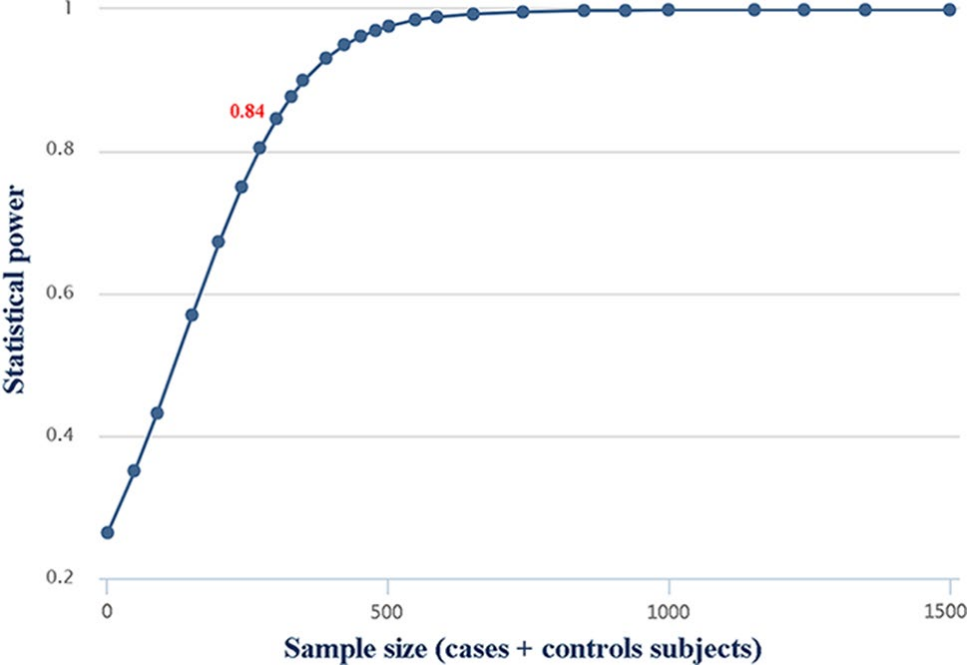
Power analysis for 1554 C > A (Ser518Arg) polymorphism with α = 0.05

According to the QUANTO program, the available sample size of cases and controls was adequate and reached the 80% threshold at the significance level of 0.05. The power of the study was calculated as 0.84 at an alpha of 0.05 (Fig. 4). This finding suggests that the rs104893674 polymorphism may contribute to the genetic predisposition of acute leukemia subtypes.

**Fig. 5:**
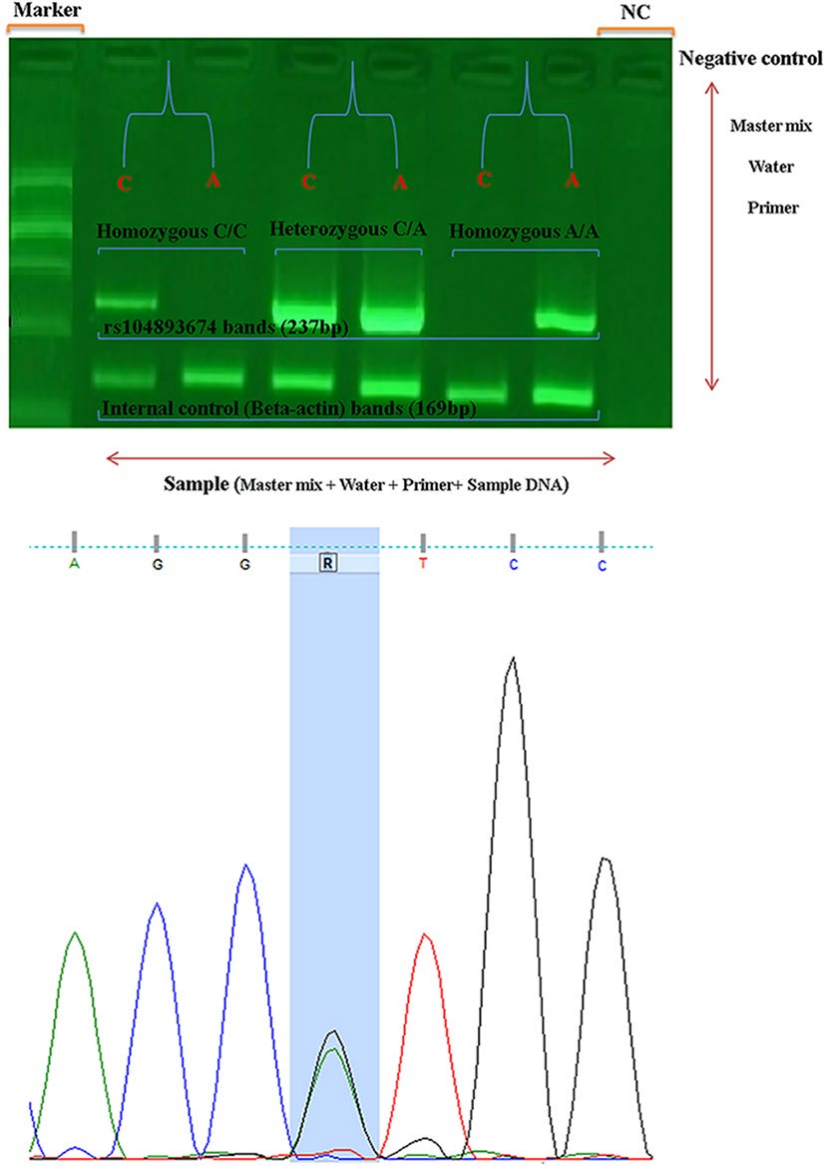
The pattern of observed bands after agarose-gel electrophoresis and result of DNA sequencing

The PCR products in Fig. 5 (Supplementary file) shown a 237 bp (rs104893674) amplification product corresponding to the allele -specific primers and a 169 bp amplification product with internal control primers when the PCR have run on a 1% agarose gel. The accuracy and specificity of our established AS-PCR was further validated by direct sequencing of PCR products.

In our study, the frequencies of the AC (F_ALL_= 89%, OR (95% CI) = 1.57(1.37–1.85), *p* < 0.004) (F_AML_= 97.1%, OR (95% CI) = 0.6(0.4–0.9), *p* = 0.031) (F _MPAL_ = 57.7%, OR (95% CI) = 0.84(0.4–1.2), *p* < 0.003) and AA (F_ALL_= 11%, OR (95% CI) = 1, *p* < 0.004) (F_AML_= 6.1, OR (95% CI) = 0.2(0.1–0.3), *p* = 0.031) (F _MPAL_ = 26.9%, OR (95% CI) = 0.46(0.15–0.3), *p* < 0.003) genotypes at rs104893674 and the A (F_ALL_= 50%, OR (95% CI) = 2.19(1.44–3.32), *p* < 0.006) (F_AML_= 54.5%, OR (95% CI) = 0.9 (0.6–0.8), *p* = 0.074) (F _MPAL_ = 96.2%, OR (95% CI) = 0.99(0.4–3.1), *p* < 0.002) allele were significantly higher in the ALL, AML and MPAL patients groups compared with controls (Table 5). Individuals with AC or AA genotypes or with the A allele at this locus were associated with an increased risk for exhibiting cancer phenotype with both myeloid and lymphoid lineages. These findings indicate the potential biological role of SNP at rs104893674 with the AC or AA genotypes and the A allele, as a common variant involved for the development of both ALL and AML. This is explained by the corresponding amino acid substitution at rs104893674 locus and would help to response to this question that; what underlying molecular features do the patients have when they observed both AML and ALL (patients who have AML and simultaneously have ALL or patients with ALL and mixed with AML)?

**Table 5:** Genotype and allele frequency distribution of rs104893674 polymorphism in ALL, AML and MPAL patients and controls

Phenotype	SNP	Controls *n* (%)	Patients *n* (%)	OR (95% CI)	Uncorrected *p*	Corrected *p*
	rs104893674				< 0.001	< 0.004
	CC	37(37.4)	0(0)	1.09(0.66–0.97)		
ALL	AC	64(62.6)	94(89)	1.57(1.37–1.85)		
	AA	0(0)	7 (11)	1	< 0.001	< 0.006
	C	136(68)	94(50)	1.47(1.20–1.80)		
	A	62(32)	94(50)	2.19(1.44–3.32)		
	rs104893674					
	CC	14(9.9)	1(1.3)	1	0.027	0.031
AML	AC	79(84)	91(97.1)	0.6 (0.4–0.9)		
	AA	2(1.6)	6(6.1)	0.2(0.1–0.3)		
	C	95(47.5)	91(45.5)	1	0.068	0.074
	A	105(52.5)	109(54.5)	0.9 (0.6–0.8)		
	rs104893674					
	CC	14(60.8)	2(15.4)	0.55(0.2–0.8)		
MPAL	AC	6(26.1)	9(57.7)	0.84(0.4–1.2)	< 0.001	< 0.003
	AA	3(13.1)	4(26.9)	0.46(0.15–0.3)		
	C	28(69.4)	4(3.8)	0.78(0.3–1.1)	< 0.001	< 0.002
	A	18(30.6)	26(96.2)	0.99(0.4–3.1)		

Note: Corrected p-values were calculated by using Bonferroni’s correction

## Discussion

The biology of co-emergence of mixed different hematopoietic lineages in MPAL has been poorly understood, and is always a matter of debate whether ALL and AML might have similar genetic traits considering their similar symptoms [14, 19]. In this study we understood the contribution of *ZAP70* gene missense mutation (rs104893674 (C > A)) in development of both lymphoid and myeloid leukemia and verified the possibility of using the rs104893674 (C > A) variant as an indicator for predicting both ALL and AML as well as mixed lineage leukemia. Identification of this common genetic factor between ALL and AML would help to uncover the points of susceptibility for MPAL and will lead to early diagnosis. With the implementation of *ZAP70* gene missense mutation (rs104893674 (C > A)) analysis in the clinical setting, a more molecularly guided precision diagnosis approach could improve the prediction accuracy for likelihood of MPAL developing. In reviewing the literature, no data was found on the association between *ZAP70* gene and/or rs104893674 SNP and mixed leukemia phenotype for providing supporting or opposing ideas, nevertheless, several lines of evidence in the literature indicate other genes that have relation to both ALL and AML. These include genes that are both mutated in the two types of leukemia [14]. Some notable are, *EP300* gene which encodes E1A binding protein p300 as a histone acetyltransferase. This protein regulates the transcription activity of many genes and plays an important role in differentiation and proliferation [20, 21]. *TRAF2* gene which is involved in the signal transduction of the TNF receptor superfamily and plays a significant role in survival and apoptosis of hematopoietic cells [22]. *JAK2* tyrosine kinase and *STAT1* both are highly associated with leukemia [23]. Mutation in *SELL* as an adhesion/homing receptor in lymphocyte–endothelial cell interactions is also responsible for the movement of blasts from bone marrow to the circulation [24]. *SELPLG* gene as a cell adhesion molecule, tethering white blood cells to the inner surface of blood vessels [25]. Generally speaking our results offer an interpretation of common genetic traits between ALL and AML. Considering a larger sample size, and likewise further studies on patients in a variety of ethnic populations as well as if an adequate population of rare MPAL patients is found, will resolve the present study limitation.

## Conclusion

MPAL often poses a diagnostic challenge owing to its rarity and underlying lineage plasticity. Identification of common genetic traits between ALL and AML would enhance our knowledge of the molecular and genetic complexity associated with MPAL to propose a novel diagnosis prediction framework.

### Electronic supplementary material

Below is the link to the electronic supplementary material.

Supplementary Material 1

## Data Availability

The datasets generated and/or analysed during the current study are available in the [dbSNP] repository [http://www.ncbi.nlm.nih.gov/SNP] and SNP can be searched for using the dbSNP ID (rs104893674).

## Abbreviations

WHO: World Health Organization
MPAL: Mixed-Phenotype Acute Leukemia
ALL: Acute Lymphoblastic Leukemia
AML: Acute Myeloid Leukemia
SNV: Single Nucleotide Variants
SAV: Single Amino Acid Variation
ZAP-70: ζ-Associated Protein Of 70 kDa
nsSNPs: Nonsynonymous Single-Nucleotide
ARMS-PCR: Polymorphisms Amplification Refractory Mutation System–Polymerase Chain Reaction
AS-PCR: Allele-Specific Polymerase Chain Reaction
PCR: Polymerase Chain Reaction
HWE: Hardy-Weinberg Equilibrium
cCD22: Cytoplasmic CD22
cCD79a: Cytoplasmic CD79a
sCD3: Surface CD3
MPO: MoAb Antimyeloperoxidase
