# Supplementary material for: Genetic evidence for predisposition to acute leukemias due to a missense mutation (p.Ser518Arg) in ZAP70 kinase: a case-control study
Source: BMC Med Genomics. 2024 Aug 7;17:200. doi: 10.1186/s12920-024-01961-0 (PMC11308335; doi:10.1186/s12920-024-01961-0)
Supplement: Supplementary file 1 — Supplementary Material 1 [file 12920_2024_1961_MOESM1_ESM.docx]

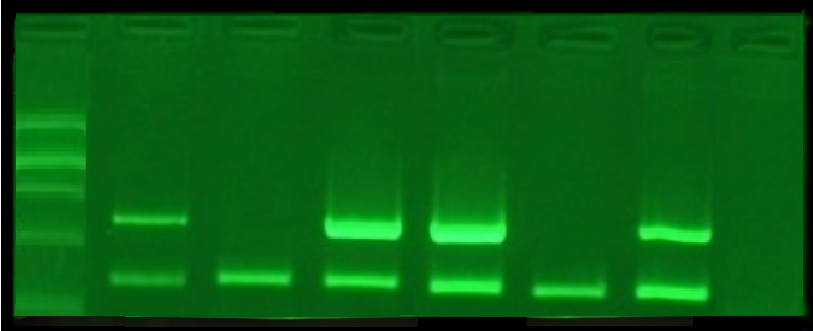


Supplementary file. Original gel with visible membrane edges including pattern of observed bands for rs104893674 SNP
